# Understanding the Physiology of Postharvest Needle Abscission in Balsam Fir

**DOI:** 10.3389/fpls.2015.01069

**Published:** 2015-11-26

**Authors:** Rajasekaran R. Lada, Mason T. MacDonald

**Affiliations:** Department of Environmental Sciences, Faculty of Agriculture, Christmas Tree Research Center, Dalhousie University, Bible Hill, NS, Canada

**Keywords:** needle abscission resistance, needle senescence, ethylene, water deficit, needle retention, *Abies balsamea*

## Abstract

Balsam fir (*Abies balsamea*) trees are commonly used as a specialty horticultural species for Christmas trees and associated greenery in eastern Canada and United States. Postharvest needle abscission has always been a problem, but is becoming an even bigger challenge in recent years presumably due to increased autumn temperatures and earlier harvesting practices. An increased understanding of postharvest abscission physiology in balsam fir may benefit the Christmas tree industry while simultaneously advancing our knowledge in senescence and abscission of conifers in general. Our paper describes the dynamics of needle abscission in balsam fir while identifying key factors that modify abscission patterns. Concepts such as genotypic abscission resistance, nutrition, environmental factors, and postharvest changes in water conductance and hormone evolution are discussed as they relate to our understanding of the balsam fir abscission physiology. Our paper ultimately proposes a pathway for needle abscission via ethylene and also suggests other potential alternative pathways based on our current understanding.

## Introduction

Balsam fir (*Abies balsamea*) trees are conifers native to northeastern North America. Stands are centralized in southeastern Canada and northeastern United States, but there are isolated populations existing as far west as Wisconsin in the United States and Alberta in Canada (Bakuzis and Hansen, 1965). Balsam fir are harvested for multiple uses, such as pulp or medicine, but it is their use as a specialty horticultural species for Christmas trees that is of most interest from a postharvest quality perspective. Balsam fir is the preferred Christmas tree species in Canada, grown on approximately 130,000 acres and worth $56 million per year (Statistics Canada, 2014). Almost 50% of all balsam fir grown in Canada are exported to other countries, such as the United States, Bermuda, Thailand, and Japan (Statistics Canada, 2014). Balsam fir is also one of the most popular species grown in the United States. A high percentage of American grown trees are also exported to a similar collection of countries as Canadian grown trees (Chastagner and Benson, 2000). Balsam fir are enjoyed due to their unique fragrance, color, and architecture.

Postharvest needle abscission is one of several challenges facing balsam fir and other conifers used as Christmas trees (Chastagner, 1986; MacDonald et al., 2010), although balsam fir is typically considered to have appreciable needle retention compared to other fir species. Chastagner and Riley (2007) had balsam fir ranked as one of the top needle retaining species, with only Noble fir and Korean fir having significantly better needle retention than balsam fir in a 3-year study. Regardless, early harvest practices combined with warmer autumn temperatures contribute to accelerated postharvest abscission. It’s been noted repeatedly that trees harvested in September or October tend to have lower needle retention than trees harvested in November or December (Mitcham-Butler et al., 1987; Chastagner and Riley, 2007; MacDonald et al., 2014b). MacDonald and Lada (2008) estimated that 1 in 3 trees shed all needles in less than 3 weeks in normal shipping/storage conditions. Such high postharvest losses emphasize the importance of understanding the abscission process.

The actual harvest itself, or root detachment, must be considered the initial stimulus for postharvest abscission. If a balsam fir tree was not cut down, then it would be unlikely to shed significant amounts of needles unless exposed to other stresses (e.g., pathogens, pests, drought). Beyond root detachment, the complete mechanisms and pathways that eventually culminate in abscission remain unknown. It has often been suggested that dehydration represents a major step toward abscission, as several studies have examined the link between critical moisture thresholds and needle loss (Hinesley and Snelling, 1997; Chastagner and Riley, 2003). But significant needle loss has also occurred even when antitranspirants were used (Chastagner and Riley, 1991; Duck et al., 2003) or when there was no discernable decrease in moisture status (MacDonald et al., 2012a). This has contributed to alternative or complimentary theories relating to the role of needle nutrition, volatile terpene compounds (VTCs), ethylene, or the lack of unknown root-derived factors no longer available after root-detachment that triggers or modulates postharvest needle abscission.

Postharvest needle abscission is a complex process, influenced by a number of external, internal, and management factors. The objective of this review is to provide insight into our current state of knowledge in postharvest abscission of balsam fir. We will discuss the dynamics of needle abscission in balsam fir while identifying key factors that modify abscission patterns, such as genotypic abscission resistance, nutrition, environmental factors, and postharvest changes in water conductance and hormone evolution. Ultimately we propose a model for postharvest needle abscission based on the information available and discuss key areas where research is still needed.

## Dynamics of Needle Abscission in Balsam Fir

**FIGURE 1:**
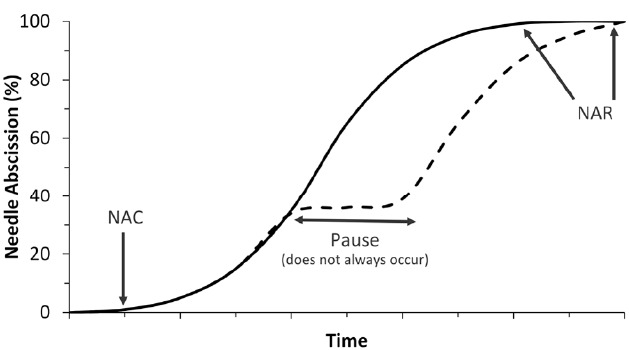
**Two most common postharvest needle abscission curves occurring in balsam fir.** The solid line denotes the typical logistic pattern of abscission while the dashed line denotes the less common pause in abscission process. Time is given no units in this diagram because although the relative curves are consistent, the timing of events is highly variable. Also marked are indication of needle abscission commencement (1% needle loss) and needle abscission resistance (100% needle loss).

The progress of postharvest needle abscission in balsam fir usually follows a logistic curve, as shown in Figure 1 (MacDonald et al., 2010, 2011b; MacDonald and Lada, 2014). After harvest and display in water, typically no abscission occurs for between one and 2 weeks. Needle abscission commencement is usually noted by a loss of 1% dry needle weight and gradually accelerates until 100% needle loss, which has been referred to as needle retention duration or, more recently, needle abscission resistance (NAR; MacDonald et al., 2014a,b). The length of time between needle abscission commencement and completion has varied between 1 week (MacDonald et al., 2011b) and 4 weeks (MacDonald et al., 2014b), though 2 weeks is a reasonable estimate in most cases.

There are instances where abscission seems to pause for a length of time before resuming. A generic version of an alternative abscission curve including a pause in abscission is shown in Figure 1 near 40% needle loss, but pauses in abscission have been observed at almost any portion of an abscission curve. For example, MacDonald et al. (2014b) observed a 2-week pause in abscission after 80% of needles had been shed before abscission resumed and eventually reached 100% needle abscission. In rare instances abscission will simply not occur or abscission will stop before reaching complete abscission and never resume (Heikkenen et al., 1986). Abscission is an energy-dependent process and if trees dehydrate too quickly then abscission cannot occur. Needles may still crumble or break off due to brittleness, but they do not truly abscise (Hinesley and Snelling, 1995).

The length of time to complete abscission has been shown to vary considerably in balsam fir, which has complicated the study of this phenomenon. In a study designed to evaluate 195 different balsam fir genotypes for NAR, it was found that some genotypes would complete abscission in as little as 6 days while others could take as long as 60 days (MacDonald and Lada, 2008). Similar results were found in a 3-year study of balsam fir, which found average NAR values between 12 and 60 days (MacDonald et al., 2014b). The variation in NAR in balsam fir led to the development of a genotype classification system where genotypes were labeled as having low NAR if they completed abscission in under 20 days, moderate NAR if they completed abscission between 20 and 40 days, and high NAR if they completed abscission in over 60 days (MacDonald, 2010). Though categorized based on needle retention, there are also certain physical differences between NAR genotypes. For instance, low NAR genotypes tend to have thicker branches, more densely distributed needles, and require more force to remove needles from fresh trees than their high NAR counterparts (MacDonald et al., 2014a). There are some physiological differences between NAR genotypes as well, which are discussed later in this article. The identification of NAR genotypes has been important as it is a major factor influencing postharvest abscission curves in balsam fir. However, the identification of a number of external and internal factors influencing postharvest abscission has been equally important.

## Environmental Factors Linked with Postharvest Abscission

The premier environmental factor to have an influence on postharvest needle abscission is cold acclimation. Cold acclimation is an adaptive process where plants acquire the ability to withstand cold temperatures through exposure to mild temperatures occurring during autumn (Gusta et al., 2005). A host of physiological changes are invoked in conifers by cold acclimation, including changes to lipid structure and composition (Senser, 1982), antioxidant molecules and enzymes (Tao et al., 1998), carbohydrates (Mitcham-Butler et al., 1987; MacDonald et al., 2014b), and abscisic acid (ABA; Thiagarajan et al., 2012, 2013). Balsam fir have been evaluated for several of these changes typically indicative of cold acclimation and were found to accumulate ABA, raffinose, and galactose in autumn months suggesting that balsam fir do undergo cold acclimation (Thiagarajan et al., 2013; MacDonald et al., 2014b). Further, needle retention also improved when subjected to a period of low temperature artificially prior to harvest (Thiagarajan et al., 2012).

Though the exact mechanism is unknown, cold acclimation generally increases postharvest needle retention in fir trees. Ten different fir species, including balsam fir, were evaluated for needle retention over 3 years and all 10 species had significantly improved postharvest needle retention in November, December, or January compared to October (Chastagner and Riley, 2007). Balsam fir had superior needle retention when harvested in November or December compared to any other month (MacDonald et al., 2014b). However, the effect of cold acclimation was not uniform on all genotypes. It was first noted by MacDonald and Lada (2008) that genotypes with poor needle retention in October tended to improve much more due to cold acclimation than genotypes with good needle retention. A comparison of needle retention in October and January harvested genotypes classified as low, moderate, and high NAR revealed that needle retention in low NAR genotypes improved by 10–15 days while there was never an improvement in high NAR genotypes (MacDonald et al., 2014b). It was speculated that high NAR genotypes may respond to environmental cues earlier than low NAR genotypes, thus high NAR genotypes may have already been acclimated in October or cold acclimation is not required for high NAR genotypes.

Photoperiod triggers a degree of cold acclimation often through a mechanism independent from temperature (Smit-Spinks et al., 1985; Welling et al., 2002). Thiagarajan et al. (2013) found that there was a significant negative relationship between photoperiod and postharvest needle retention in balsam fir in two different genotypes. MacDonald et al. (2014b) did not include a discussion of photoperiod in their study, but an examination of needle retention each month suggests that there would at least be a relationship with the reported average monthly needle retentions and photoperiod. Such a relationship was described in a subsequent study where photoperiod was shown to have more influence that temperature in modifying needle retention (MacDonald and Lada, 2015). The above studies all found that a low photoperiod improves postharvest needle retention, which also corresponds with the superior needle retention observed in November or December harvested trees (MacDonald et al., 2014b).

Adjustments to postharvest environmental conditions, specifically lighting and vapor pressure deficit, also affected needle retention. Balsam fir branches stored in darkness or provided fluorescent lighting (253.7 μmol·s^–1^·m^–2^) had a NAR of 42.3 days and 38.0 days, respectively. However, exposure to white, red, or blue light emitting diodes significantly improved needle retention by over 50% (Veitch et al., 2012). Modifying vapor pressure deficit had an even greater impact on needle retention. When balsam fir branches were stored at vapor pressure deficits greater than 0.9 kPa, then NAR was 32 days or less. When branches were stored a vapor pressure deficits less than 0.9 kPa, then NAR was significantly increased. For example, at 0.23 kPa NAR had increased to 150 days (MacDonald et al., 2012a).

## Preharvest Nutrition Linked with Postharvest Abscission

Preharvest nutrition has been a relatively minor area of study, though has shown a definitive impact on postharvest needle abscission in balsam fir. There is little standardization on fertilizer application rates, with only some emphasis placed on nitrogen (Georgeson, 2013). Most nutrients had no link to postharvest needle abscission. However, branches with higher foliar nitrogen, potassium, copper, and iron all had accelerated postharvest needle abscission (Georgeson, 2013). Nutrients were all present at concentrations lower than necessary to induce toxicity, thus the mode of action to explain a negative relationship with needle abscission remains unknown. Elevated ethylene evolution rate is one potential explanation, as iron and copper have each been linked to ethylene in other species (Mapson and Wardale, 1968; Ben-Yehosua and Biggs, 1970). External application of several nutrients also had minimal effect on postharvest needle abscission, though zinc significantly accelerated abscission (Georgeson, 2013).

One potential limitation in the work conducted by Georgeson (2013) was that experiments were often done with only one or two specific genotypes, in an effort to control experimental error. However, this also provided a more narrow range of foliar nutrient concentrations. The work from Georgeson (2013) was repeated in a natural tree stand, as opposed to clonal orchard. Several similar relationships were found, but higher concentrations of soil and foliar calcium were associated with delayed postharvest abscission (MacDonald and Lada, 2015). It is speculated that higher endogenous concentrations of foliar calcium delayed abscission by improving stability of cell walls and impeding cell wall degradation in abscission zones (Xu et al., 2009). The role of preharvest nutrient in needle abscission could be further explored by focusing on cell wall changes in the abscission zone.

## Postharvest Changes to Water Consumption and Hydraulic Conductivity

Water deficit and dehydration is an immediate postharvest concern in balsam fir. A freshly cut balsam fir tree consumes approximately 0.15–0.20 mL·g^–1^·d^–1^ and has a stomatal conductance of 20–25 mmol·m^–2^·s^–1^, but both rapidly decrease after harvest (MacDonald and Lada, 2014; Lada et al., 2015a; MacInnes, 2015). The first response is a decrease in stomatal conductance, which decreases by 50% within 4 days of harvest and decreases by 80% within the first week (MacInnes, 2015). The change in water consumption is slightly slower than the change in stomatal conductance, but within 2 weeks it is not uncommon for water consumption to decrease to 0.05 mL·g^–1^·d^–1^ (MacDonald and Lada, 2014; Lada et al., 2015a). Postharvest needle abscission in balsam fir typically begins when water consumption falls below 0.05 mL·g^–1^·d^–1^, which suggests any improvement to water status or water uptake may delay abscission (Lada et al., 2015a; MacDonald and Lada, 2015). The precise cause of decreased water consumption is not yet known, though cavitation, embolism, stomatal dysfunction, bacteria contamination, or blockage of xylem vessels have all been speculated.

Postharvest water status has been studied extensively in many root-detached conifers (Bates et al., 2004). Water status is conventionally assessed by percent moisture content, relative water content, or xylem pressure potential (XPP) of the branches. XPP is likely the most often used and is an indicator of tension at which water is held in the xylem conduits. Threshold values have been established for several conifers, which represent the XPP from which a tree is unable to recover and results in accelerated needle loss, discoloration, and a tendency to defoliate even under rehydration (Hinesley and Snelling, 1991). For example, the range in which trees could successfully rehydrate was –4.0 to –5.0 MPa in *Leyland cypress* (Hinesley and Snelling, 1995), –4.0 to –4.5 MPa in Fraser fir (Hinesley, 1984), and approximately –3.0 MPa in nordmann fir (Chastagner and Riley, 2003). Rehydration in balsam fir has not been studied as extensively as some other species, but the threshold in which rehydration may successfully occur is approximately 45% moisture content (Adams et al., 2013). As noted in earlier sections of the review, balsam fir inherently has a high degree of variation in its postharvest characteristics. The moisture content from which balsam fir could successfully rehydrate was also highly variable, though it was linked with NAR. Balsam fir genotypes characterized as high NAR could successfully rehydrate from a water content as low as 38% while balsam fir genotypes categorized as low could not rehydrate from moisture contents below 47% (Adams et al., 2013).

Postharvest needle abscission has occurred in several studies when XPP has been maintained above –1.0 MPa, which is not indicative of water stress (MacDonald et al., 2012a,b; MacDonald and Lada, 2014). However, though the final XPP values during abscission were not exceptionally low in those studies, they were significantly lower than fresh XPP values. Further, there were other studies where XPP fell as low as –6.0 MPa, which would indicate water stress (Lada et al., 2015a). XPP has not had a strong relationship with needle abscission in some fir species (Bates et al., 2004), but there have been significant relationships with needle abscission in balsam fir (MacDonald et al., 2012a,b). Other evaluators, such as relative water content or percent moisture, all consistently decrease after harvest leading to abscission (MacDonald et al., 2012a; MacDonald and Lada, 2014) and there was a strong relationship between moisture content and postharvest needle abscission (MacDonald and Lada, 2015). Overall, there was consistently a decrease in water status in postharvest balsam fir that was highly linked to abscission.

Efforts to mitigate decrease in water status have a significant positive effect in limiting balsam fir needle abscission. Lada et al. (2015a) identified decreasing water quality in Christmas tree stands as having an adverse effect on needle retention, possibly due to an exponential increase in bacterial counts. When water was routinely drained and replaced with fresh water, then NAR was increased by 38%. Conversely, when water that was previously drained from a Christmas tree stand was provided to a freshly cut tree, then there was a 36% decrease in NAR (Lada et al., 2015a). An alternative method to maintain water status was to store branches in a low vapor pressure deficit environment, which effectively maintained XPP and relative water content at fresh harvest values. Storage at low vapor pressure deficit increased NAR fivefold (MacDonald et al., 2012a). Finally, a study was conducted that mounted balsam fir branches on a simulated root pressure system that could maintain water flow by generating positive pressure. Low levels of positive pressure were sufficient to delay abscission (MacInnes, 2015).

It is important to note that although a decrease in water status is a major factor that accelerates needle loss, hydration alone cannot retain needles indefinitely. Postharvest needle abscission still ultimately occurred in situations where water status was maintained through changes to water delivery, modifying vapor pressure deficit, or applying antitranspirants (Duck et al., 2003; MacDonald et al., 2012a; MacInnes, 2015). There must be a physiological signal that triggers abscission due to water stress, but also a signal that triggers abscission even if there is no water stress. The signal could be the same in both instances or could be triggered through different pathways. Ethylene triggers abscission in many species (Brown, 1997) and is a candidate for inducing postharvest abscission in balsam fir through one of the aforementioned pathways.

## Ethylene as a Key Signal for Postharvest Needle Abscission

Ethylene, the simplest unsaturated hydrocarbon, is a plant hormone often produced in response to stress in many species, including conifers. For example, ethylene evolution was significantly increased in jack and white pines due to drought (Rajasekaran and Blake, 1999; Islam et al., 2003), in silver fir due to biotic stresses (Fuhrer, 1985), and Norway spruce due to ozone and drought stress (Van den Driessche and Langebartels, 1994). Though ethylene is involved in a host of physiological processes, ethylene evolution due to stress is often associated with senescence and abscission as a defense response (Brown, 1997).

Ethylene evolution began slowly after harvest, but reached a peak several weeks after harvest in several conifers (Alvarez-Moctezuma et al., 2007). The pattern of ethylene evolution was very similar in balsam fir, with almost no detectable ethylene in the few days and then reaching a peak after several weeks (MacDonald et al., 2010). Due to evidence suggesting that ethylene in conifers is triggered via water stress (Rajasekaran and Blake, 1999), it is quite possible that deteriorating water status of postharvest balsam fir causes ethylene evolution in balsam fir. Interestingly, the progression of ethylene evolution increased in parallel with needle abscission, ultimately reaching its peak 1–3 days before complete abscission (MacDonald et al., 2010). Korankye (2013) also noted increased ethylene evolution along with other volatiles that were associated with abscission. Ethylene evolution has also been associated with genotypic differences in balsam fir needle retention; low NAR genotypes released significantly higher concentrations of ethylene and had accelerated abscission due to lower concentrations of exogenous ethylene (MacDonald et al., 2012b). Thus, endogenous ethylene increased in balsam fir and was, at the very least, associated with postharvest abscission.

**FIGURE 2:**
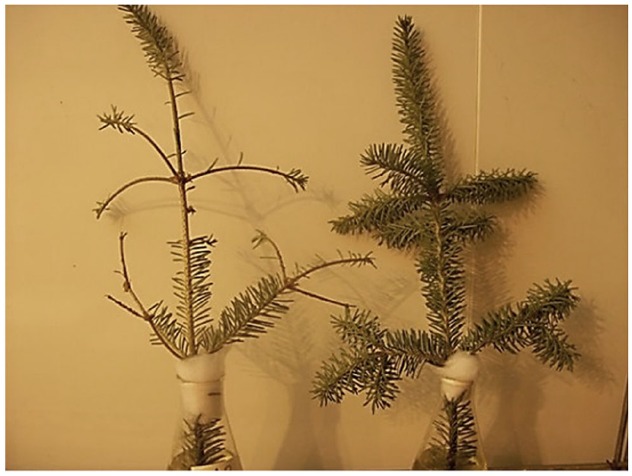
**Comparison of a balsam fir branch after prolonged exposure to 1000 ppm ethylene (left) to a branch stored in the absence of ethylene (right) after 14 days (**MacDonald et al., 2011a**)**.

There is strong evidence that ethylene is not only associated with abscission, but actually induces abscission in balsam fir (Figure 2). Prolonged exposure to exogenous ethylene has consistently decreased needle retention by 60–70% (MacDonald et al., 2010, 2011a,b). Exogenous ethylene also accelerated abscission uniformly regardless of NAR genotype (MacDonald et al., 2012b) and regardless of storage temperature or humidity (MacDonald et al., 2012a). Further, ethylene inhibition with aminoethoxyvinylglycine and 1-methylcyclopropene delayed abscission. Aminoethoxyvinylglycine inhibits ethylene synthesis by blocking the conversion of S-adenosyl-L-methionine to 1-aminocyclopropane-1-carboxylic acid (Boller et al., 1979). Xylem feeding aminoethoxyvinylglycine to balsam fir delayed abscission by up to 113%, though abscission was still accelerated 60–70% in the presence of exogenous ethylene (MacDonald et al., 2010). Conversely, 1-methylcyclopropene blocks the effect of ethylene by competitively binding to ethylene receptors (Sisler and Serek, 1997). Foliar application of 1-methylcyclopropene to balsam fir delayed abscission by 73% in the absence of exogenous ethylene and delayed abscission by 118% in the presence of exogenous ethylene compared to respective controls (MacDonald et al., 2010). The only exception to exogenous ethylene inducing abscission in balsam fir was found in a study exploring the effect of acute ethylene exposure. Acute ethylene exposure actually helped to delay abscission, though the mechanism for this has not been explored (MacDonald et al., 2011b).

Ethylene is believed to facilitate abscission by promoting the production of cell wall hydrolytic enzymes, such as cellulase, in the abscission zone (Tucker et al., 1988). This mode of action may also occur in balsam fir, as there was an eightfold increase in cellulase activity due to endogenous ethylene exposure and a 12-fold increase in cellulase activity due to exogenous ethylene exposure (MacDonald et al., 2011a). It remains unknown whether ethylene may also stimulate production of other hydrolytic enzymes in balsam fir which may contribute to abscission.

The role of ethylene in postharvest abscission has practical implications as preharvest handling by industry includes shaking and baling of Christmas trees, which may act as a form of mechanical stress. One concern is that mechanical stress is associated with ethylene synthesis and abscission in many species (Mitchell and Myers, 1995), thus it is conceivable that normal Christmas tree handling processes contribute to postharvest abscission in balsam fir. There is a lack of information available in this area, but it has been shown that baling is linked to accelerated abscission patterns (Korankye et al., 2015). Non-baled balsam fir trees lost the least percentage of needles postharvest compared to any baled treatment, with some links to volatile terpene and ethylene evolution (Korankye et al., 2015). There is ongoing research in the area of preharvest handling and its link to abscission.

## Involvement of Other Plant Hormones in Balsam Fir Abscission

In addition to ethylene, ABA is likely the most studied hormone with respect to postharvest abscission in balsam fir. The concentration of ABA in fresh balsam fir need tissue was 350 ng·g^–1^ to 500 ng·g^–1^ (Thiagarajan et al., 2012; MacDonald and Lada, 2014). However, during abscission there is a 39-fold increase in ABA concentrations and even higher increase in ABA metabolites (Table 1; MacDonald and Lada, 2014). The increase in ABA during abscission has raised the possibility that ABA may be a trigger for abscission. Zhang et al. (2009) found that application of exogenous ABA application induced ethylene evolution and abscission. Likewise, use of the ethylene inhibitor silver thiosulfate in conjunction with ABA negated any ABA-induced abscission (Suttle and Abrams, 1993). These results suggest ABA could be an intermediary messenger for ethylene in the balsam fir abscission pathway. However, ABA is also well known to facilitate stomatal closure in response to drought (McAdam et al., 2011), which means that the increase in balsam fir ABA during abscission could simply be an artifact of postharvest water deficit.

**TABLE 1:** **Summary of known changes in phytohormones in fresh balsam fir compared to actively abscising balsam fir. Ethylene data is from MacDonald et al. (2010) and remaining hormone data is from MacDonald and Lada (2014)**.

**Hormone**	**Fresh concentration**	**Abscising concentration**	**Change**
Ethylene (μL·g^–1^·d^–1^)	0.5	15.1	30-fold increase
ABA (ng·g^–1^)	342	13,263	39-fold increase
Cytokinins (ng·g^–1^)*	51	143	3-fold increase
Auxins (ng·g^–1^)	99	5	20-fold decrease

*cytokinins represent the total of trans-zeatin-O-glucoside, trans-zeatin riboside (dominant species), cis-zeatin riboside, dihydrozeatin riboside, isopentenyl adenosine, and isopentenyl adenine.

Abscisic acid may also be associated with needle retention due to ABA’s involvement with cold acclimation. MacDonald et al. (2014b) observed a twofold increase in foliar ABA and Thiagarajan et al. (2012) observed a fourfold increase in foliar ABA due to cold acclimation. The relative increase in ABA due to cold acclimation is much lower than the postharvest increase in ABA, but it was speculated that the increased ABA may play a role in the improved needle retention after cold acclimation. There was some support to this theory when exogenously supplied ABA at very low concentrations resulted in improved needle retention in balsam fir, while higher concentrations of ABA promoted needle abscission (Thiagarajan, 2012).

Auxins also appear to play a role in postharvest abscission of balsam fir. The predominant auxin in balsam fir is indole-3-acetic acid (IAA), which was detected 99 ng·g^–1^ in freshly harvested balsam fir (Table 1; MacDonald and Lada, 2014). However, there was a 95% decrease in IAA after harvest (MacDonald and Lada, 2014). Of interest is the antagonistic relationship between ethylene and auxin; IAA is considered to inhibit abscission while ethylene is thought to promote abscission. The general rule portrays that provided the flux of IAA to the abscission zone is maintained, then cell separation is inhibited and abscission does not occur (Taylor and Whitelaw, 2001). The postharvest decrease in balsam fir IAA is likely linked to ethylene-induced abscission.

Cytokinins are conventionally believed to delay abscission. As plants begin to senesce, cytokinin concentrations in the leaves and sap show a marked decrease (Noodén et al., 1990; Singh et al., 1992) and exogenous application of cytokinins on senescing tissue has demonstrated the ability to delay or reverse the process in some species (Adedipe and Fletcher, 1971). Further, endogenous cytokinins in root and needle tissue have a strong, positive correlation with needle retention (MacDonald and Lada, 2015). Roots are believed to be the major source of cytokinin synthesis (Chen et al., 1985), thus it reasons that root-detachment via harvest would impair cytokinin synthesis and contribute to abscission. However, postharvest changes in cytokinins exhibited the opposite response as expected (Table 1). Six species of cytokinins were identified in balsam fir needles, with trans-zeatin riboside contributing to 50% of the overall cytokinin concentration, and all species of cytokinins increased during abscission (MacDonald and Lada, 2014). As noted above with respect to auxins, the changes in cytokinins were noted during abscission compared to initial fresh concentrations, which means it would be beneficial to understand exactly when the concentration of cytokinins increased with respect to abscission.

## Volatile Terpene Compounds and Their Role in Postharvest Needle Abscission

Ethylene, through a variety of approaches, is shown to be a key signal involved in postharvest abscission of balsam fir (MacDonald et al., 2010). However, abscission still ultimately occurs in situations where ethylene is inhibited (MacDonald et al., 2010, 2012a), which has suggested that an ethylene-independent pathway must exist for postharvest needle abscission.

Volatile terpene compounds constitute a large class of secondary compounds produced by conifers which contribute to their characteristic smell (Schmelz et al., 2003). VTCs are most commonly associated with plant defense (Croteau et al., 2000), but may be linked to ethylene evolution and abscission. A total of 12 VTCs have been detected in balsam fir, which all have a tendency to increase postharvest corresponding with needle abscission (Korankye, 2013). When ethylene was inhibited, needle retention was delayed but eventually occurred following an increase in volatile terpene concentrations (Korankye, 2013). The concentration of volatile terpenes released was also correlated with percentage needle loss in baled versus non-baled trees, despite no significant difference in ethylene evolution rates (Korankye et al., 2015). It is speculated that volatile terpenes could be part of an ethylene-independent abscission pathway, though more work is needed to test that theory.

## Postharvest Changes in Membrane Stability, Chlorophyll, and Lipid Profiles

Membranes are often one of the first sites of drought damage in conifers (Rajasekaran and Blake, 1999) and, in fact, are often deteriorated in response to any form of abiotic stress (Yang and Hoffman, 1984). The exact mechanisms are not fully understood, but it is generally accepted that stresses trigger the generation of free radicals and reactive oxygen species which in turn trigger lipid peroxidation, increased permeability of cellular membranes, and senescence (Droillard et al., 1987; Hodges et al., 2004). Harvest procedures, postharvest storage, water loss, and ethylene all contribute to oxidative stress and membrane damage in many species (Hodges et al., 2004). Thus, it is logical to suggest the aforementioned postharvest factors could induce oxidative stress in balsam fir. Further, lipid peroxidation is thought to contribute to ethylene synthesis (Paulin et al., 1986; Pell et al., 1997), which suggests reactive oxygen species and changes to membrane integrity could be a key step in the needle abscission pathway.

Measurements of the changes in reactive oxygen species or endogenous antioxidants in postharvest balsam fir have not been performed. However, there have been some measurements to assess changes in membrane stability. The main method of evaluating membrane stability in balsam fir has been to calculate the membrane injury index, which essentially reports a ratio of the amount of electrolytes leaking from collected needles to the total electrolytes present (Rajasekaran and Blake, 1999). Membrane injury index of balsam fir does not change in autumn when trees experience colder temperatures, which suggests a level of membrane protection due to cold acclimation that may or may not be linked to superior needle retention (Thiagarajan et al., 2013; MacDonald et al., 2014b). Immediately after harvest, membrane injury was relatively low and remained low for almost 3 weeks. However, during abscission there was a 50% increase in membrane injury in balsam fir (MacDonald and Lada, 2014). Similar results were found in a different study, where membrane injury was relatively low until a 134% increase leading to abscission (MacDonald et al., 2015). The delay in membrane injury between harvest and abscission may be due to little discernable water stress since relative water content and XPP also remained relatively high for the first 3 weeks postharvest (MacDonald and Lada, 2014). As of yet, it remains undetermined whether membrane injury is a cause or symptom of abscission.

Membrane injury is apparent in chloroplasts of balsam fir. The chlorophyll index of postharvest balsam fir begins relatively high, but gradually decreases until abscission (MacDonald and Lada, 2014). Chlorophyll fluorescence is one tool to assess photosynthetic activity in a plant, chloroplast stability, and general physiological status of a plant (Ball et al., 1995). Postharvest fluorescence decreases more slowly in conifers than many other species (Richardson and Berlyn, 2002; Fangyuan and Guy, 2004), but there is a distinct relationship between fluorescence and needle abscission in balsam fir. Similar to changes in chlorophyll index, fluorescence gradually decreases postharvest until abscission. However, there is a very strong negative relationship (R^2^ > 90%) between fluorescence and needle abscission, which suggests at the very least that fluorescence and chloroplast integrity are associated with postharvest needle abscission (MacDonald and Lada, 2015). Further, galactolipids found primarily in the chloroplast (i.e., monogalactosyldiacylglycerol and digalactosyldiacylglycerol) significantly decreased during abscission, which also suggests chloroplast membrane breakdown associated with abscission (MacDonald et al., 2015). The relationship between membrane damage and onset of ethylene evolution has yet to be explored in balsam fir, but membrane protection via antioxidant application has suppressed ethylene evolution and decreased membrane leakage in other conifers (Borsos-Matovina and Blake, 2001; Islam et al., 2003).

## Summary of Postharvest Abscission in Balsam Fir and Future Research

**FIGURE 3:**
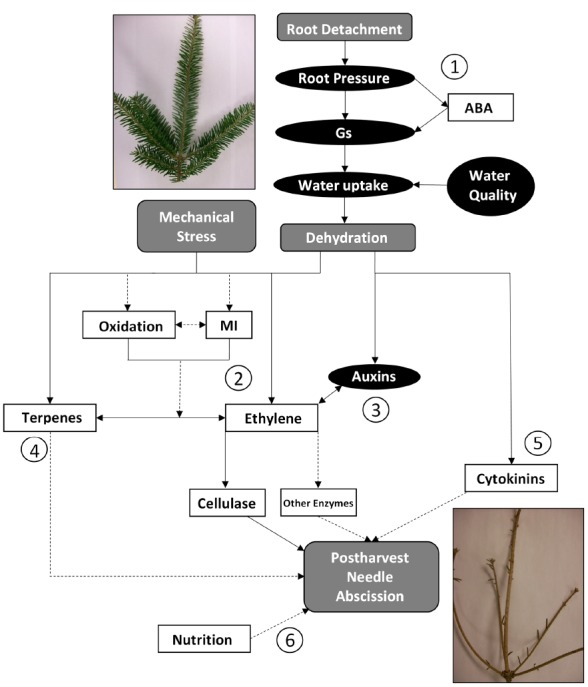
**Proposed pathways for postharvest needle abscission in balsam fir.** Elements in gray represent events which are known to occur, elements in black ovals represent factors which decrease, and elements in white squares represent factors which increase. The circled numbers represent key portions and/or areas requiring more research. Dashed arrows are used to indicate portions of the pathway that are speculative or have only weak supporting evidence to date. Gs: stomatal conductance; ABA: abscisic acid; MI: membrane injury.

Postharvest needle abscission in balsam fir is a complex physiological event involving many different factors. Cold acclimation (Thiagarajan et al., 2013; MacDonald et al., 2014b), genotypic variability (MacDonald, 2010), soil and needle nutrition (Georgeson, 2013), volatile terpenes (Korankye, 2013), water status (Lada et al., 2015a; MacInnes, 2015), light (Veitch et al., 2012), vapor pressure deficit (MacDonald et al., 2012a), ethylene (MacDonald et al., 2010), and other hormones (MacDonald and Lada, 2014; Lada et al., 2015b) all influence postharvest abscission in some manner. The proposed pathway for postharvest needle abscission in balsam fir is shown in Figure 3. There are six points of discussion labeled and research needed to further develop the theory is discussed below:

1. Root detachment during harvest is the initial trigger that leads to a decrease in root pressure, stomatal conductance, water content, and XPP (MacInnes, 2015; Lada et al., 2015a). There is also a postharvest increase in ABA, which may induce stomatal closure in an effort to conserve water (Thiagarajan et al., 2012). Transpiration is the driving force for water uptake in a branch, thus it is logical to suggest that decreased stomatal conductance leads to decreased water uptake (MacInnes, 2015). It has been shown that deteriorating water quality or bacteria accumulation in water supply can exacerbate the situation (Lada et al., 2015a). Ultimately, it is generally agreed that an immediate consequence of root detachment is dehydration.
2. The major pathway to postharvest balsam fir abscission is via ethylene (MacDonald et al., 2010, 2011a,b, 2012a,b; Korankye, 2013). Ethylene then triggers cellulase, which most likely weakens cell walls in the abscission zone to facilitate abscission (MacDonald et al., 2011a). It is hypothesized that other hydrolytic enzymes (i.e., polygalacturonase) are involved in degrading cell walls to perpetuate abscission. Further, understanding the order of postharvest events, such as oxidation, membrane injury, and ethylene synthesis is a key area of research to further develop the proposed pathway. Do reactive oxygen species cause membrane damage and then induce ethylene? Or is membrane damage another symptom of increased ethylene evolution?
3. Auxins decrease by 95% in balsam fir leading to abscission (MacDonald and Lada, 2014) and application of exogenous auxins, such as naphthalene acetic acid, delayed abscission in balsam fir (Lada et al., 2015b). The main area of research needed in this area is a temporal investigation into changes in endogenous auxins after harvest, which may then be compared to changes in ethylene synthesis and needle abscission.
4. It is not yet known whether volatile terpenes are a cause or a result of postharvest abscission. Are they part of a general defense response induced by harvest, or do they function as a complimentary or alternative pathway to ethylene? VTCs increased after harvest (alongside ethylene) and are highest in high NAR genotypes (Korankye, 2013). Both endogenous and exogenous volatile terpenes were associated with abscission when ethylene was inhibited (Korankye, 2013). Whether volatile terpenes induce abscission requires further study, though it is hypothesized that jasmonic acid could be involved. The role of jasmonic acid in regulating volatile terpenes and/or ethylene pathways should be explored.
5. Cytokinins are typically believed to delay senescence and abscission, but cytokinins increased two- to fourfold during abscission of balsam fir (MacDonald and Lada, 2014). It was once speculated that cytokinins, or rather lack of root-derived cytokinins, could contribute to postharvest abscission and could ultimately be an abscission pathway independent of dehydration (MacDonald et al., 2012a). That may still be a possible pathway, but there is a clear gap in our understanding of cytokinins. A temporal understanding of postharvest cytokinin changes in balsam fir would represent a major contribution to our understanding. It may be that cytokinins only increase at the very end of senescence to allow for some cell division or separation in the abscission zone. Or perhaps the increase in cytokinins represents a defensive reallocation of resources. Very little information is available regarding development of abscission zone in balsam fir, which may be linked to increases in postharvest cytokinins. Another useful contribution would be to investigation the effect of exogenous cytokinin application or manipulation of endogenous cytokinins through inhibition or genotypic investigations.
6. There is some evidence that foliar nitrogen, potassium, copper, and iron increased, then postharvest needle retention decreased in one study (Georgeson, 2013). The impact, mode of action, or interaction with other abscission-modifying factors has not yet been explored.

A complete understanding of the processes governing postharvest needle abscission is necessary to develop methods to halt or delay abscission. The abscission curve is described as a logistic curve and it is probably not realistic to believe postharvest needle abscission can be prevented. However, treatments that shift the abscission curve to the right would represent a method of delaying or inhibiting abscission. Referring again to Figure 3, anything that targets a major step in the postharvest balsam fir abscission pathway should, in turn, mitigate abscission. This has been observed by applying positive pressure to negate the effects of decreasing root pressure postharvest (MacInnes, 2015), providing clean water to maintain water uptake and prevent xylem blockage (Lada et al., 2015a), storage in high vapor pressure deficit to delay dehydration (MacDonald et al., 2012a), or inhibit ethylene to maintain cell wall stability in abscission zones (MacDonald et al., 2010). All aforementioned methods significantly delayed abscission, which not only contribute to our scientific understanding and validation of the abscission pathway, but also represent potential abscission mitigating technologies.

## Author Contributions

RL and MM each contributed to gathering the literature for review, writing the initial draft of the article, and conceptual design of intellectual content. RL and MM reviewed the manuscript independent from each other before meeting to decide which revisions to adopt. Both RL and MM approved the final article for submission and agree to be held accountable for all aspects of the work.

### Conflict of Interest Statement

The authors declare that the research was conducted in the absence of any commercial or financial relationships that could be construed as a potential conflict of interest.
